# Novel Polymorphic Microsatellite Loci for the Korean Black Scraper (*Thamnaconus modestus*), and Their Application to the Genetic Characterization of Wild and Farmed Populations

**DOI:** 10.3390/ijms12064104

**Published:** 2011-06-20

**Authors:** Hye Suck An, Eun Mi Kim, Jang Wook Lee, Chun Mae Dong, Bai Ik Lee, Yi Cheong Kim

**Affiliations:** 1 New Strategy Research Center, National Fisheries Research and Development Institute, Busan 619-705, Korea; E-Mails: bilee@nfrdi.go.kr (B.I.L.); yckim@nfrdi.go.kr (Y.C.K.); 2 Biotechnology Research Division, National Fisheries Research and Development Institute, Busan 619-902, Korea; E-Mails: ocean0329@nfrdi.go.kr (E.M.K.); ehdcnsao@nfrdi.go.kr (C.M.D.); 3 Genetics and Breeding Research Center, National Fisheries Research and Development Institute, Gyeongsangnamdo 656-842, Korea; E-Mail: lee9952@nfrdi.go.kr

**Keywords:** Korean black scraper, *Thamnaconus modestus*, microsatellite loci, genetic marker, genetic variability

## Abstract

In this study, we developed 20 polymorphic microsatellite markers for the Korean black scraper, *Thamnaconus modestus* (Günther, 1877), Monacanthidae, and used them to compare allelic variation between wild and hatchery populations in Korea. All loci were readily amplified and demonstrated allelic variability, with the number of alleles ranging from 5–35 in the wild population and 5–22 in the farmed population. The average observed and expected heterozygosities were estimated, respectively, as 0.74 and 0.80 in the hatchery samples and 0.78 and 0.81 in the wild ones. These results indicate lower genetic variability in the hatchery population than in the wild population and minor, but significant, genetic differentiation between the two populations (*F*_ST_ = 0.005, *P* < 0.01). Additionally, cross-amplification was tested in another monacanthid species, *Stephanolepis cirrhifer*; many loci were found that yielded useful information. The high degree of polymorphism exhibited by the 20 microsatellites will be useful in future aquaculture and population genetic studies for developing conservation and management plans.

## 1. Introduction

The black scraper, *Thamnaconus modestus* (Günther 1877), Monacanthidae, is a tropical oceanodromous fish found in the Indo-West Pacific Ocean, from the Korean Peninsula, Japan, and China Sea to East Africa. It inhabits coastal waters, feeding primarily on plankton and benthic organisms and is cultured commercially in Japan and Korea [1]. In Korea, total catches of ~230,000 tons of black scraper were harvested annually until 1990, since when the commercial catch has declined continuously. While the causes of the decline are unknown, habitat loss from coastal development and overfishing may have contributed. The catch decline and increased consumption have increased interest in black scraper artificial breeding practices and genetic studies for a sustainable fishery. Understanding genetic structure patterns is increasingly important for developing effective fishery conservation strategies, management, and remediation efforts. Understanding genetic divergence and diversity is important in black scraper aquaculture for selective breeding.

Using molecular and biotechnological tools, the genetic background of black scraper populations can be understood. Microsatellite (MS) DNA markers are useful in population genetics studies and stock evaluations; they are powerful tools for assessing genetic diversity in fishes and the typically high number of alleles at these loci could make them particularly sensitive to detecting inbreeding in aquaculture populations [2–4]. Many MS markers must be developed and screened to identify a collection of loci that provide efficient population genetics analyses. Despite the high commercial interest in Korean black scraper, no specific MS marker has yet been characterized. Furthermore, no reported study has examined their genetic variability or population structure.

In this study, we developed 20 novel polymorphic MS primer sets from an enriched *T. modestus* DNA library to support future genetic studies and examined differences in the genetic variability at these loci between wild and hatchery populations. Additionally, the applicability of these markers in another monacanthid species was evaluated via cross-species amplification.

## 2. Results and Discussion

### 2.1. Microsatellite Loci Isolation

In total, >500 white colonies were obtained from transformation with the Korean black scraper (CA)*_n_*-enriched genomic DNA library. Of these, each small portion of about 300 colonies was tested for a repeat-containing insert using PCR. Sequencing the inserts from 150 insert-containing colonies revealed 71 loci containing microsatellite arrays with a minimum of five repeats, corresponding to an enrichment efficiency of 47.3%. These were primarily 2-bp repeat motifs, some of which were combined with other 2-bp repeat motifs. The primers were designed and tested for 55 loci that exhibited adequately long and unique sequence regions flanking the microsatellite array. Of these, only 20 primer sets, 36.4%, successfully yielded variable specific PCR products: KTm138, KTm141, KTm151, KTm25, KTm220, KTm222, KTm223, KTm234, KTm246, KTm252, KTm254, KTm255, KTm261, KTm268, KTm271, KTm274, KTm279, KTm283, KTm286, and KTm292. The remaining 35 primer sets (63.6%) gave either inconsistent or no PCR products, despite adjusting the dNTP concentrations and using an annealing temperature gradient. The primer sequences, repeat motifs, annealing temperatures, fluorescent labels, and GenBank accession numbers for the 20 new microsatellite loci are summarized in Table 1.

Traditionally, MS DNA loci isolation has relied on screening genomic libraries using repetitive probes and sequencing positive clones to develop locus-specific primers. This method is tedious, but can acquire numerous MS DNA loci. Magnetic-bead-based enrichment is a common method for constructing MS-enriched libraries. The types and ratios of biotin-labeled probes and the positive clone selection strategy can affect cloning success and enrichment efficiency. In this study, we created MS libraries enriched for CA repeat sequences using the protocol of Hamilton *et al.* [5] and modifications described by Gardner *et al.* [6] and Carleton *et al.* [7]. Of the positive clones obtained, about 47.3% (71/150) contained MS repeats; less than in rockfish at 50% [8], flounder at 74% [9], and tilapia at 96% [7], but higher than in Japanese Spanish mackerel at 34% [10]. The enrichment efficiency differences probably resulted from using various biotin-labeled oligonucleotide probes and the proper ratio rather than different absolute numbers of repeats in each genome.

### 2.2. Genetic Characterization

Understanding the genetic diversity of black scraper populations is vital for stock abundance recovery and planning sustainable fishery management. Microsatellite DNA loci are expected to be invaluable because their highly polymorphic characteristics have great potential as genetic tags. Thus, we identified and characterized the first reported set of microsatellite markers for the Korean black scraper *T. modestus*.

Samples of 60 wild and 30 hatchery-bred *T. modestus* collected from Geoje, Korea, were screened for variation at the 20 new polymorphic MS loci. The 20 primer sets yielded variable profiles. Reruns were conducted for 40% of all individuals to ensure allele scoring reproducibility.

The MICRO-CHECKER analysis revealed that some loci could have been influenced by one or more null alleles in both the wild and hatchery samples; our data demonstrated that loci KTm151, KTm25, and KTm271 in the farmed samples and loci KTm271 and KTm286 in the wild population were affected. The locus KTm271 appeared to be influenced by null alleles in both the wild and hatchery samples, indicating that it could be problematic for population genetic analyses that assume Hardy-Weinberg equilibrium. Thus, global multilocus *F*_ST_ values were estimated with and without this locus. For KTm151, KTm25, and KTm286, different factors indicated that these loci were affected by null alleles in only one sample; thus, they were included in further analyses.

No genotyping errors from allele dropouts or stuttering affected the allele scoring. Samples that failed to amplify after the rerun were not included, making it unlikely that poor DNA quality affected the results.

In total, 295 alleles were observed in the 20 loci; the number of alleles per locus varied from 4 at KTm271 to 35 at KTm246 (Table 2). The overall allelic richness varied from 4 to 28.13 (Table 2). The wild population had more alleles and greater allelic richness than did the hatchery-bred population, although the difference was not statistically significant (*P* > 0.05). The observed heterozygosity ranged from 0.100 at KTm25 to 0.967 at KTm222, KTm223, KTm246, KTm252, and KTm254, whereas the expected heterozygosity varied from 0.160 at KTm25 to 0.968 at KTm246 (Table 2). In this study, high genetic diversity (mean heterozygosity =0.81; mean allelic number =10.53) was detected in the wild population, slightly higher than reported in most other marine fishes [11]. Although the genetic diversity of *T. modestus* is relatively high, populations are probably declining. Thus, the genetic diversity of *T. modestus* should be protected.

The inbreeding coefficients (*F*_IS_) varied among markers, from −0.250 at KTm141 to 0.381 at KTm25 in the hatchery samples and from −0.194 at KTm254 to 0.335 at KTm271 in the wild samples. The wild population had more unique alleles and a higher frequency of the most common allele than the hatchery population (Table 2).

Significant departures from HWE after the Bonferroni correction (*P* < 0.003) were found at two loci, KTm271 and KTm286, in the wild population, indicating that deviations from HWE were due to heterozygote deficiency. Significant heterozygote deficiency has previously been reported in other marine fishes [12,13]. The presence of null alleles is a locus-dependent effect found frequently at MS DNA loci. Null alleles most likely cause heterozygote deficiency in HWE tests [14]. The MICRO-CHECKER analysis in this study revealed null alleles at KTm271, which had a significant heterozygote deficit.

Because this study was limited by the number of populations screened, the genetic diversity parameters for each population and the HW disequilibrium at KTm286 in the wild samples might be explained by data from additional populations, which would provide more precise estimates for the genetic characterization of the MS loci. Thus, our results should be interpreted with caution. A timely, systematic study is required to assess wild population genetic resources and the influence of aquaculture on the genetic structure of this species.

**Figure 1:**
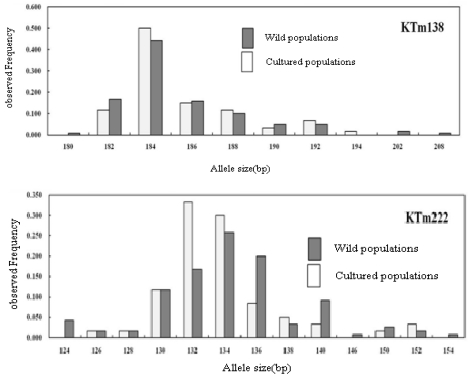

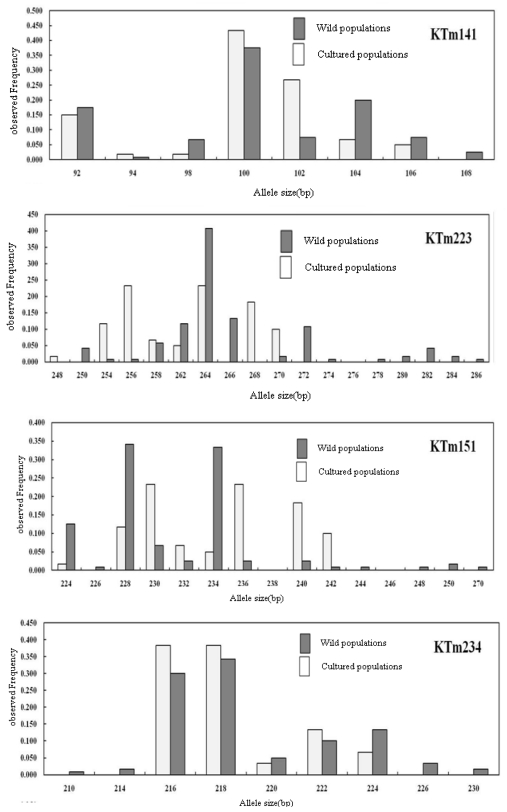

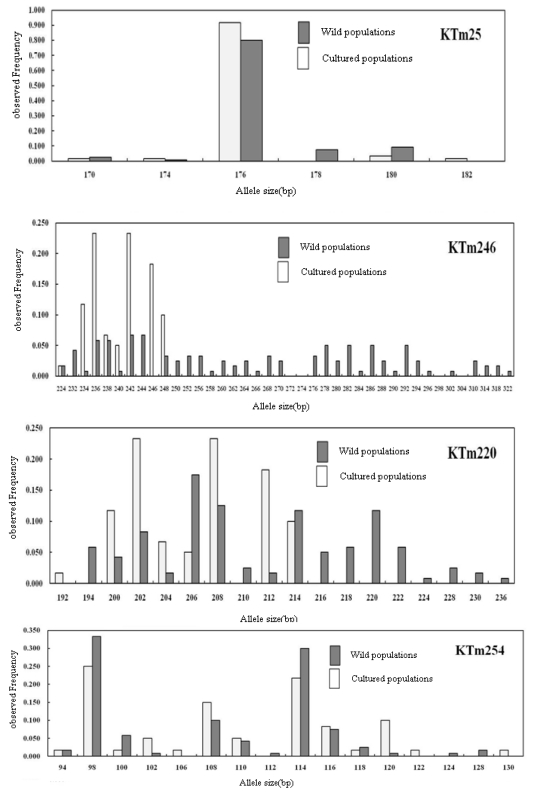

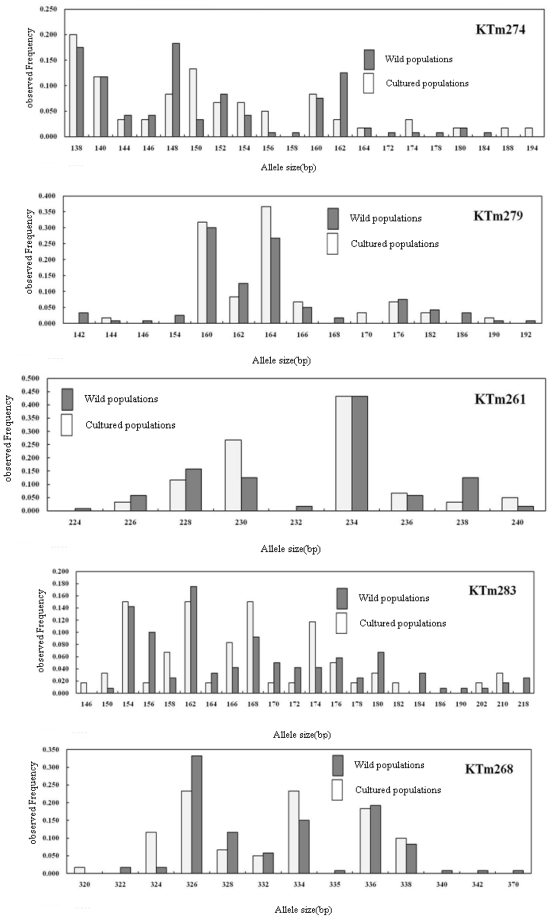

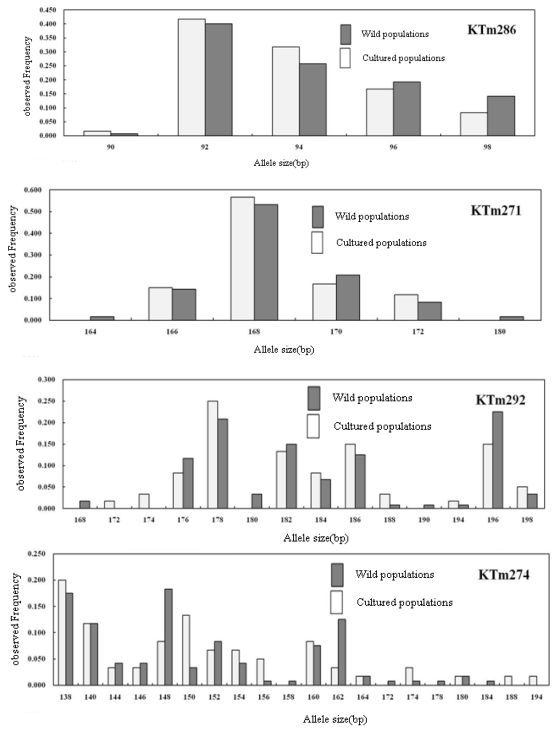
Allele size frequency distributions of the 20 microsatellite loci of *Thamnaconus modestus* used in this study.

The allele frequencies of the 20 microsatellites in the wild and hatchery samples are listed in Figure 1. A homology search using the program BLAST showed that none of the 20 sequences were similar to any GenBank sequence. Rare alleles with a frequency <5% were detected at most loci. Examination of linkage disequilibrium for all pairs of loci using a likelihood-ratio test by ARLEQUIN version 3.0 [15] revealed that all the six microsatellite loci were in linkage equilibrium (*P* > 0.003).

The global multilocus *F*_ST_ values differed significantly between the hatchery and wild populations. It was estimated to be 0.005 (*P* < 0.01). When locus KTm271 was excluded, the global multilocus *F*_ST_ was estimated to be 0.006 (*P* < 0.01). The wild and hatchery populations had minor, but significantly different, global multilocus *F*_ST_ values. The significant *F*_ST_ estimates indicate genetic differentiation between the populations, probably as a result of reduced genetic variation. In Geoje, Korea, the black scraper progeny produced for stock abundance recovery was different in genetic composition, although no significant reduction was found in the mean heterozygosity or diversity compared with the wild population (*P* > 0.05; Table 2). The genetic variability was lower in the hatchery population than in the wild population, which can be attributed to a low effective number of founding individuals in the hatchery population, with the effects of artificial selection on hatchery progeny. Several studies have reported a loss of genetic variation at MS loci in hatchery populations and a reduced fitness in hatchery-bred individuals when exposed to natural environments [16,17]. Reduced genetic variation can decrease aquaculture performance, because this is a source of variation for important traits, such as growth rate and disease resistance [18,19]. Moreover, allele loss is more important than a change in allele frequency, as the latter can be changed again by random drift, whereas lost alleles cannot be recovered. To manage commercial breeding programs properly, genetic structure and diversity must be monitored, in addition to biological, ecological, and fishery factors.

### 2.3. Cross-Species Amplification

Additionally, cross-species amplification of primers was screened in another monacanthid species, *Stephanolepis cirrhifer*. 12 of the primer pairs effectively amplified, showing consistent polymorphisms. The number of alleles per locus ranged from four to eight. The characteristics of these 12 primer sets are presented in Table 3.

Cross-species amplification is effective only if primer sequences are conserved between species. The monacanthids studied in this experiment are the most important filefish fishery resources in Korea. In this study, 12 pairs of primers amplified in *S. cirrhifer* and the number of alleles obtained for each locus in *S. cirrhifer* differed from those of *T. modestus*. Generally, the number of amplified loci tends to decrease in proportion to increasing divergence between species [20,21].

## 3. Experimental Section

### 3.1. Library Construction and Sequencing

To construct a genomic DNA library, the TNES-urea buffer method [22] was used to isolate high-molecular-weight DNA (20 μg) from fin tissue of an individual black scraper from Geoje, Korea.

A partial genomic library, enriched for CA repeats, was constructed using a modified enrichment procedure with pre-hybridization polymerase chain reaction (PCR) amplification, as described previously [5]. The extracted DNA was digested with the restriction enzymes *Alu*I, *Rsa*I, *Nhe*I, and *Hha*I (New England Biolabs, USA). Then, 300- to 800-bp-long DNA fragments were isolated and purified using a QIAquick Gel Extraction Kit (QIAGEN, Germany). The selected fragments were ligated to an adaptor (SNX/SNX rev linker sequences) and the linker-ligated DNA was amplified using SNX as a linker-specific primer for PCR. For enrichment, the DNA was denatured and the biotin-labeled repeat sequences (CA)_12_GCTTGA [23] were hybridized to the PCR products. The hybridization complex was removed using streptavidin-coated magnetic spheres (Promega, USA). After the complex was washed, the bound, enriched DNA was eluted from the magnetic spheres and re-amplified with an adaptor sequence primer. PCR products were then purified using the QIAquick PCR Purification Kit (QIAGEN, Germany).

The purified PCR products were digested with *Nhe*I, cloned using an *Xba*I-digested pUC18 vector (Pharmacia, USA), and transformed into *Escherichia coli* DH5α competent cells. White colonies were screened for a repeat insert using PCR with the universal M13 primer and non-biotin-labeled dinucleotide primers. PCR products were examined on 2% agarose gels and inserts producing two or more bands were considered to contain an MS locus. Positive clones were cultured and purified. Plasmids from the insert-containing colonies were recovered using the QIAprep Spin Miniprep Kit (QIAGEN, Germany) and sequenced using the BigDye Terminator Cycle Sequencing Ready Reaction Kit (ver. 3.1; Applied Biosystems, USA) and an automated sequencer (ABI Prism 310 Genetic Analyzer; Applied Biosystems).

### 3.2. Primer Design and Genotyping

Primers were designed based on sequences flanking the MS motifs using the OLIGO software package (ver. 5.0; National Biosciences, USA). Newly designed PCR primer pairs were tested to optimize the annealing temperatures; a gradient PCR with a 50–60 °C range was performed on a sample set from eight black scrapers captured from Geoje, Korea. The PCR amplification was performed using a PTC 200 DNA Engine (MJ Research, USA) in a 10-μL reaction containing 0.25 U of *Ex Taq* DNA polymerase (TaKaRa Biomedical, Japan), 1 × PCR buffer, 0.2 mM dNTP mix, 100 ng of template DNA, and 10 pmol of each primer, where the forward primer from each pair was 5′-end-labeled with 6-FAM, NED, and HEX dyes (Applied Biosystems). The PCR reaction ran for 11 min at 95 °C, followed by 35 cycles of 1 min at 94 °C, 1 min at the annealing temperature (Table 1), and 1 min at 72 °C, with a 5-min final extension at 72 °C. Microsatellite polymorphisms were screened using an ABI PRISM 3100 Automated DNA Sequencer (Applied Biosystems) and alleles were designated by PCR product size relative to a molecular size marker (GENESCAN 400 HD [ROX], Applied Biosystems). Fluorescent DNA fragments were analyzed using the GENESCAN (ver. 3.7) and GENOTYPER (ver. 3.7) software packages (PE Applied Biosystems).

### 3.3. Sample Comparisons

In December 2008, 60 black scrapers were captured off the coast of Geoje, Korea. In May 2008, 30 farmed samples were obtained from a hatchery-reared population used as broodstock for artificial reproduction in Geoje, Korea. Although the hatchery population had been reared continuously, their origins and records were unavailable. For genotyping, total DNA was extracted using a MagExtractor-genomic DNA purification kit (TOYOBO, Japan) following the manufacturer’s recommendations for the automated MagExtractor MFX–2100 DNA extraction system (TOYOBO, Japan). The extracted genomic DNA was stored at −20°C until used for PCR.

Samples were screened for variation at the newly developed MS loci. MICRO-CHECKER 2.2.3 [24] was used to detect genotyping errors due to null alleles, stuttering, or allele dropout using 1000 randomizations. For genetic diversity parameters, the number of alleles per locus (*N*_A_), size of alleles in base pairs (*S*), frequency of the most common allele (*F*), and number of unique alleles (*U*) were determined for each local sample at each locus using the program GENEPOP (ver. 4.0; http://kimura.univ-montp2.fr/~rousset/Genepop.htm). This was also used to identify deviation from Hardy-Weinberg equilibrium (HWE; exact tests, 1000 iterations) and the observed and expected heterozygosities, indicating an excess or deficiency of heterozygotes. FSTAT version 2.9.3.2 [25] was used to calculate the inbreeding coefficient (*F*_IS_) [26] per locus and sample and allelic richness (*A*_R_) [27], suitable for comparing the mean number of alleles among populations regardless of sample size. ARLEQUIN 3.0 [15] was used to assess linkage disequilibrium for all loci pairs [28] and to calculate single-locus and global multilocus values (*F*_ST_; 1000 permutations) [26]. Significance levels were adjusted for multiple tests using sequential Bonferroni correction [29].

## 4. Conclusions

In conclusion, we report, for the first time, the development of 20 polymorphic microsatellite markers that can be used to detect *T. modestus* population structures. Moreover, 12 of the microsatellite markers in *T. modestus* can be used in the related species *S. cirrhifer*. These markers can be used in future studies on population genetics, conservation genetics, and fishery management of these two monacanthids.

## Figures and Tables

**Table 1:** Characteristics of the 20 microsatellite loci isolated from *Thamnaconus modestus*.

Locus	Repeat Motif	Primer Sequence (5′→3′)	*T*_a_ (°C)	Genbank Accession No.
KTm138	(CA)_13_-(CA)_8_	F: CTAATTTCCCAAGGTAAGGTT **6-fam** R: TCATTAAAAAGCCAATCAGAA	60	FJ210983
KTm141	(GT)_12_	F: CGTGATGTCGCTGTAAGG **hex** R: TCTGGCAGTTTTTCTTTTTAT	57	FJ210984
KTm151	(GT)_22_	F: CACAGACAGAAGGTCAGAGAG **6-fam** R: ATTCATGCAACTTACACGACT	55	FJ210987
KTm25	(GT)_6_-(GT)_5_	F: TGATTACCTCAAAACTTGTGT **hex** R: CCTTCAGCTGTAAATCTCATA	57	FJ210993
KTm220	(GT)_7_CT(GT)_7_AT(GT)_10_	F: AGTTTTTAGATTTGCGGTTGT **6-fam** R: AATTCCCCCTACAGTCTTCTC	57	FJ210997
KTm222	(GT)_13_	F: TGGGTTTTGTTGGGTAA **hex** R: CCCCCTCTATTTAGATTATGT	57	FJ210999
KTm223	(CA)_11_CT(CA)_9_	F: CTGCTGCACGTGTCTCA **ned** R: TGTGATAGCCAAAGTCTGATG	57	FJ211000
KTm234	(CA)_14_	F: TTGCCTTTACAGTGACAAACA **6-fam** R: GGATGGAGGGGAAGAGA	63	FJ211001
KTm246	(CA)_12_	F: AAACGGCTCATGTTAATCTGT **ned** R: AATTCTGCAGCCGTTTAGAC	63	FJ211003
KTm252	(GT)_24_	F: ACACGTTTGCAGATTTGTAAT **hex** R: GCCACTCTCTAGGGTAGTAGG	57	FJ211006
KTm254	(GT)_13_	F: AGCCCTAATATAAACACACTG **hex** R: TGCGCAGGATACATTG	52	FJ211007
KTm255	(GT)_11_	F: AGCGAAGAGAACATTCCTC **ned** R: CTGCCAAGATCCTAACTTTGT	57	FJ211008
KTm261	(GT)_17_	F: TTCAGATTTGATTGTGAGGAT **6-fam** R: TGAAGGGCAGACTTGTTTAC	63	FJ211010
KTm268	(GT)_10_	F: ATTATCACCCCCACAAGTTCT n**ed** R: CGGCTAAATCATGTTTCTGA	60	FJ211012
KTm271	(GT)_16_	F: TGTGGTGTTTACTGCAGATAA **hex** R: ACAGGGTCATGAAAATAATGT	57	FJ211013
KTm274	(GT)_20_	F: ATGGAAATAAGCCTCTTGTTC **hex** R: CTTCTGCAAACCTAAATCAAA	57	FJ211016
KTm279	(GT)_5_TT(GT)_15_	F: AGTTTGACGGCTGACATTTAT **hex** R: AGCGCTTCAGATCAGATTCT	57	FJ211018
KTm283	(CA)_35_	F: GATCTCCATCTCCACCTT **6-fam** R: TTGGCTATTGGTAAATTATTC	57	FJ211019
KTm286	(CA)_12_	F: GAACTGTGCAACTGTGTTTTC **hex** R: AAATGTCGCTGTATCTGCTG	63	FJ211021
KTm292	(GT)_14_	F: ATCTGCCATTCACTTCCTTTA **hex** R: CGGAATGTGATCTGCTTTG	51	FJ211022

*T*a is the optimal annealing temperature.

**Table 2:** Summary of the statistics for the 20 microsatellite loci in the two *Thamnaconus modestus* populations.

Population (No)	Microsatellite Loci																					
		KTm 138	KTm 141	KTm 151	KTm 25	KTm 220	KTm 222	KTm 223	KTm 234	KTm 246	KTm 252	KTm 254	KTm 255	KTm 261	KTm 268	KTm 271	KTm 274	KTm 279	KTm 283	KTm 286	KTm 292	Mean
	*F_S_*_T_	−0.007	0.028	0.025	0.026	0.018	0.017	0.002	−0.002	0.010	−0.006	0.008	−0.001	0.009	0.007	−0.013	0.006	−0.002	0.002	−0.011	−0.006	0.005
**Geoje Wild (60)**	*N*_A_	9	8	13	5	17	13	15	9	35	18	13	16	9	12	6	18	14	20	5	12	12.44
	*A*_R_	7.73	7.37	9.88	4.38	14.95	11.05	11.69	7.93	28.13	14.42	10.35	14.18	7.99	9.50	5.50	14.36	11.47	17.16	4.50	10.13	10.53
	*S*	180–208	92–108	224–270	170–180	194–236	124–154	250–286	210–230	224–322	104–144	94–128	320–368	224–240	322–370	164–180	138–184	142–192	150–218	90–98	168–198	
	*F*	0.442	0.375	0.342	0.800	0.175	0.258	0.408	0.342	0.067	0.175	0.333	0.258	0.433	0.333	0.533	0.183	0.300	0.175	0.400	0.225	0.32
	*U*	3	1	4	1	5	3	5	4	18	4	3	5	2	5	2	4	6	4	0	3	3.4
	*H*_E_	0.743	0.779	0.756	0.348	0.911	0.846	0.789	0.768	0.968	0.898	0.783	0.879	0.755	0.812	0.650	0.894	0.817	0.918	0.722	0.854	0.811
	*H*_o_	0.750	0.750	0.733	0.333	0.917	0.800	0.800	0.767	0.967	0.900	0.933	0.933	0.750	0.867	0.433	0.917	0.867	0.950	0.500	0.800	0.780
	*F*_IS_	−0.010 (0.990)	0.037 (0.040)	0.030 (0.044)	0.043 (0.587)	−0.007 (0.131)	0.055 (0.724)	−0.015 (0.501)	0.001 (0.739)	0.002 (0.525)	−0.002 (0.579)	−0.194 (0.005)	−0.063 (0.302)	0.006 (0.740)	−0.068 (0.593)	0.335 (0.000)	−0.025 (0.924)	−0.062 (0.043)	−0.035 (0.650)	0.310 (0.000)	0.064 (0.300)	
	*P*	0.984	0.039	0.064	0.621	0.127	0.661	0.541	0.698	0.600	0.547	0.008	0.304	0.756	0.639	0.000	0.903	0.033	0.683	0.000	0.352	
**Geoje Hatchery (30)**	*N*_A_	7	7	11	5	13	10	13	5	22	17	13	12	7	8	4	16	9	18	5	11	10.00
	*A*_R_	7.00	7.00	11.00	5.00	13.00	10.00	13.00	5.00	22.00	17.00	13.00	12.00	7.00	8.00	4.00	16.00	9.00	18.00	5.00	11.00	10.00
	*S*	182–194	92–106	224–250	170–182	192–230	126–152	248–282	216–224	232–304	104–146	94–130	324–368	226–240	320–338	166–172	138–194	144–190	146–210	90–98	172–198	
	*F*	0.500	0.433	0.233	0.917	0.167	0.333	0.367	0.383	0.117	0.200	0.250	0.317	0.433	0.233	0.567	0.200	0.367	0.150	0.417	0.250	0.33
	*U*	1	0	2	1	1	0	3	0	5	3	3	1	0	1	0	2	1	2	0	2	1.0
	*H_E_*	0.706	0.723	0.860	0.160	0.915	0.786	0.795	0.694	0.950	0.897	0.859	0.831	0.731	0.841	0.625	0.913	0.759	0.915	0.703	0.870	0.799
	*H*_o_	0.767	0.900	0.700	0.100	0.933	0.967	0.967	0.600	0.967	0.967	0.967	0.800	0.733	0.833	0.467	0.867	0.700	0.933	0.567	0.800	0.744
	*F*_IS_	−0.087 (0.635)	−0.250 (0.096)	0.189 (0.090)	0.381 (0.002)	−0.020 (0.431)	−0.235 (0.003)	−0.220 (0.480)	0.138 (0.103)	−0.018 (0.603)	−0.080 (0.123)	−0.128 (0.116)	0.038 (0.524)	−0.004 (0.902)	0.009 (0.031)	0.257 (0.187)	0.052 (0.342)	0.079 (0.674)	−0.021 (0.010)	0.196 (0.158)	0.082	
	*P*	0.640	0.091	0.051	0.004	0.447	0.004	0.464	0.114	0.685	0.181	0.989	0.135	0.539	0.907	0.035	0.199	0.390	0.663	0.014	0.132	

Single-locus *F*_ST_, number of samples (*N*_o_), number of alleles per locus (*N*_A_), allellic richness (*A*_R_), size in bp of alleles (*S*), frequency (*F*) of the most common allele, number of unique alleles (*U*), expected heterozygosity (*H*_E_), observed heterozygosity (*H*_o_), inbreeding coefficient (*F*_IS_), and probability of significant deviation from Hardy-Weinberg equibrium after Bonferroni correction (*P*, initial α = 0.05/20 = 0.003) are given for each population and locus. Calculations assume that individuals with one microsatellite band are homozygous for the allele. Number in parenthesis below *F*_IS_ indicates the probability of significant heterozygosity excess or deficit.

**Table 3:** Cross-species amplification of Stephanolepis cirrhifer using *Thamnaconus modestus* microsatellite primers.

Species (No)	Microsatellite Loci												
		KTm 141	KTm 151	KTm 220	KTm 222	KTm 234	KTm 252	KTm 254	KTm 255	KTm 261	KTm 274	KTm 279	KTm 292
***Step hanolepis cirrhifer*(20)**	*N*_A_	6	7	8	7	6	6	4	6	6	7	5	7
	*S*	92–106	228–244	206–228	132–186	216–230	106–130	94–120	328–368	216–240	138–154	138–170	172–196
	*T*a (°C)	52	57	57	52	52	57	52	63	63	57	57	52

Number of samples (*N*_o_); number of alleles per locus (*N*_A_), size of alleles in bp (*S*), and annealing temperature (*T*_a_) are given for the amplified loci.
